# MM-NIDS: A Novel Multimodal Ensemble Fusion Network Intrusion Detection System Using Numeric, Text, Graph, and Quantum Representations

**DOI:** 10.3390/s26134196

**Published:** 2026-07-02

**Authors:** Samar AboulEla, Rasha Kashef

**Affiliations:** Electrical, Computer, and Biomedical Engineering, Toronto Metropolitan University, Toronto, ON M5B 2K3, Canada

**Keywords:** cybersecurity, network intrusion detection, transformers, language models, graph embedding, quantum encoding, deep learning, multimodal

## Abstract

The proliferation of digital infrastructures and the Internet of Things (IoT) has led to a rapid increase in interconnected devices, exposing modern systems to increasingly sophisticated cyber threats. Intrusion detection in such environments remains a major challenge due to limited device resources, evolving attack vectors, and diverse traffic patterns. Traditional systems often fall short in scalability and adaptability when facing these modern threats. This paper introduces MM-NIDS, a novel multimodal fusion framework for NetFlow-based intrusion detection. The framework combines four complementary NetFlow-derived data representations (numerical, textual, graph-based, and quantum-inspired), each modeled using transformer-based architectures, including FT-Transformer and ELECTRA-Small. Feature embeddings are constructed using robust engineering techniques, while predictions from the four base models are integrated through five post hoc fusion strategies: averaging-based fusion, weighted averaging, confidence-based fusion, and two meta-fusion methods based on a Multi-Layer Perceptron (MLP) and Extreme Gradient Boosting (XGBoost). Extensive cross-dataset evaluations on four public NetFlow-based benchmarks confirm the system’s robustness, with the text-based model (M2) consistently achieving the highest individual performance. Fusion approaches provided modest and dataset-dependent improvements in detection balance, especially for underrepresented attacks. A detectability hypothesis was proposed and validated, showing that NetFlow features are particularly effective for volumetric and scan-based attacks but less so for stealthy, payload-driven threats. These findings highlight the potential of MM-NIDS for deployment in critical infrastructure, industrial IoT, and smart environments, suggesting that future work should incorporate deeper semantic or payload-level features to enhance the detection of evasive threats further.

## 1. Introduction

Intrusion detection is a cornerstone of cybersecurity, involving continuous monitoring of network traffic to identify unauthorized access, malicious behaviors, and policy violations. As network environments grow in complexity and scale, particularly with the proliferation of Internet of Things (IoT) devices, the need for intelligent, context-aware detection systems has never been greater. Traditional rule-based solutions struggle to adapt to evolving threats, driving research toward adaptive, machine learning-driven approaches that can discern subtle patterns and anomalies in real time.

To design and evaluate intrusion detection systems (IDS), it is useful to consider established detection taxonomies. The National Institute of Standards and Technology (NIST) [1] identifies three primary IDS approaches: signature-based detection, which matches incoming traffic against known attack fingerprints; anomaly-based detection, which flags deviations from learned normal behavior; and stateful protocol analysis, which inspects protocol semantics for compliance violations. These approaches provide complementary perspectives for recognizing known attacks, deviations from normal behavior, and protocol misuse. Within this broader taxonomy, this work focuses on flow-level intrusion detection using NetFlow records and positions the proposed MM-NIDS framework as a data-driven IDS approach.

The relevance of this direction is reinforced by the rapid growth of IoT environments, which has introduced new cybersecurity challenges [2,3,4] by expanding the attack surface across billions of devices with limited resources, from sensors to smart appliances. These devices often lack the computational power and energy reserves needed for resource-intensive IDS operations. In addition, IoT environments generate massive, heterogeneous data streams, including encrypted traffic, that challenge real-time analysis. Persistent issues such as high false positive rates, limited scalability, and deployment complexity hinder traditional IDS effectiveness, motivating the development of robust flow-based detection frameworks that can analyze compact traffic summaries such as NetFlow records.

Maintaining confidentiality, integrity, and availability (CIA) [5] requires early and accurate detection of threats such as data breaches, unauthorized access, and denial-of-service attacks. Although machine learning (ML) and deep learning (DL) have improved IDS performance, many models still face high false positives, limited adaptability to emerging attacks, and weak generalization across environments. They may also miss contextual and semantic signals, such as protocol misuse or payload intent, which are important for detecting sophisticated or stealthy threats.

Large language models (LLMs) offer a promising avenue for enriching intrusion detection with semantic and contextual understanding. Their ability to process structured and unstructured data, ranging from NetFlow logs to tokenized payloads and graph representations, makes them well-suited for multimodal threat analysis. However, applying transformer-based models to NetFlow-based intrusion detection requires careful alignment between representation design, detection performance, and computational cost. In this work, we focus on improving detection robustness and class-wise attack visibility through the fusion of complementary NetFlow-derived representations.

This paper presents a novel multimodal, ensemble-based IDS framework that incorporates numerical, textual, graph-based, and quantum-inspired features, leveraging transformer architectures (FT-Transformer and ELECTRA-Small) and a suite of fusion strategies, including averaging, weighted averaging, confidence-based fusion, and meta-fusion using MLP and XGBoost. We validate our approach on four public NetFlow (NF) datasets (BoT-IoT, ToN-IoT, UNSW-NB15, CSE-CIC-IDS2018), evaluate an attack detectability hypothesis, and optimize critical parameters to balance performance and resource usage.

The novelty of MM-NIDS lies in the unified integration and fusion of four complementary NetFlow-derived representations: numerical, textual, graph-based, and quantum-inspired encodings. Rather than treating these branches as independent sensing modalities, this work uses them as complementary views of the same NetFlow records. In addition, we introduce a NetFlow attack detectability hypothesis and evaluate it through class-wise diagnostic analysis to explain why some attack categories are more observable from flow-level features than others.

Despite being based on the identical NetFlow fields, the four modalities capture fundamentally diverse aspects of the data, allowing the system to discover complementary patterns that a single encoding cannot provide. The numeric modality retains basic statistical features for direct feature-level modeling, while the text modality includes semantic links by translating flows into sentence-like sequences, allowing ELECTRA to leverage contextual dependencies. The graph modality provides inter-sample relational information via k-NN similarity graphs and Node2Vec embeddings, while the quantum modality introduces nonlinear correlations through qubit-based angle embeddings with superposition-like interactions. When combined, these diverse representations enrich the feature space and improve the robustness of the multimodal intrusion detection framework. These heterogeneous encodings induce distinct inductive biases, which is the sense in which this work uses the term “multimodal.”

The remainder of the paper is organized as follows: Section 2 reviews related work; Section 3 outlines the key background; Section 4 details our methodology; and Section 5 presents a multi-tiered experimental study, starting with a global performance evaluation across four benchmark datasets. Following that, a class-by-class diagnostic assessment is conducted using a proposed ‘Detection Meter’ to assess specific attack detectability. Lastly, to show the resilience of the framework, we conduct a thorough sensitivity analysis and hyperparameter adjustment. The paper concludes in Section 6 with a discussion of evaluation insights, limitations, and future research directions.

## 2. Related Work

This section reviews prior intrusion detection systems (IDSs) organized into three categories: ML/DL-based methods, transformer-based approaches, and hybrid or explainable AI techniques. For each, we outline representative techniques, datasets used, and their limitations.

### 2.1. ML- and DL-Based IDS

Classical ML methods such as Decision Trees (DTs) and Support Vector Machines (SVMs) rely on manual feature engineering and struggle with imbalanced data [6]. In contrast, deep learning (DL) models (e.g., Multi-Layer Perceptrons (MLPs) and Long Short-Term Memory (LSTM)) can automatically extract features from raw network flows. A stacking ensemble described in [7] uses GAN-based sampling on NSL-KDD dataset to mitigate class imbalance and improve detection accuracy. Su et al. [8] propose BAT-MC, combining BiLSTM with an attention mechanism for multiclass classification on NSL-KDD, but report high false negatives. Mazumder et al. [9] fuse k-means clustering with ensemble learners (XGBoost, RF, LightGBM) to enhance performance at the expense of longer training times. Xu et al. [10] employ a five-layer autoencoder on NSL-KDD, achieving 90.61% accuracy but finding that deeper models offer diminishing returns. GAN-based resampling methods, including GAN-RF [11], GAN-ECNN [12], and Bi-GAN [13], also address imbalance, but face detection gaps or computational overhead. Packet-level early detection via GAN-augmented LSTM-DNN [14] and classic k-NN/RF/SVM classifiers [15] demonstrates rapid inference but often trades off overall accuracy or transparency.

### 2.2. Transformer-Based IDS

Transformer-based and LLM approaches utilize contextual embeddings to identify anomalies in network traffic. Fu et al. [16] introduce IoV-BERT-IDS for vehicular networks, pretraining with byte sentence tasks, next byte sentence prediction (NBSP) and masked byte word model (MBWM), to generalize with limited labels, yet struggle to distinguish similar attacks in real time. Long et al. [17] apply a standard transformer encoder for cloud security, achieving high accuracy against DDoS and botnets but demanding significant resources. Hassanin et al. [18] present PLLM-CS for satellite IDS, capturing long-range dependencies while lacking domain-specific benchmarks. Liu et al. [19] enhance transformers with autoencoder positional encoding and hybrid sampling, thereby improving multiclass accuracy while maintaining computational efficiency. Wang et al. [20] propose TabTransformer for tabular traffic data, outperforming classical ML yet limited by deployment complexity. A lightweight transformer-based IDS [21] employs compact models (e.g., BERT-tiny, DistilBERT) fine-tuned on IoT traffic, balancing efficiency and accuracy but with limited scalability and generalization. Proactive prediction with GPT, BERT, and LSTM fusion [22] and BERT-based contextual analyzers [23] achieves up to 98% accuracy but incurs misclassification or overhead issues. Resource-efficient variants utilize PEFT/PPO quantization on Llama 2 [24] and knowledge-distilled CNN students [25]. In contrast, federated BERT in 5G contexts [26] and systematic LLM evaluations [27] highlight trade-offs between scalability, zero-day generalization, and computational cost.

### 2.3. Hybrid and Explainable AI Approaches

Hybrid and explainable AI (XAI) approaches aim to combine multiple paradigms for interpretability and robustness. HuntGPT [28] integrates RF anomaly detection with SHAP/LIME explanations and GPT-3.5 for interactive insights, yet it is validated only on KDD99 and may not scale. ShieldGPT [29] uses GPT-4 for DDoS detection and mitigation advice, offering rich explanations but relying on manual instruction validation. Qin et al. [30] fuse knowledge graphs with ML to dynamically model threats from logs and CTI data, improving interpretability while incurring high computational costs. Ghosh et al. [31] propose ASGAFGNN, combining ViT, BiLSTM, guided-attention GNN, and federated learning for spatio-temporal anomaly detection, achieving strong accuracy under privacy constraints but requiring extensive preprocessing.

Table 1 consolidates the reviewed IDS methods, datasets, and limitations across ML/DL, transformer-based, and hybrid XAI approaches. Overall, while ML/DL, LLM-based, and hybrid XAI methods have advanced detection performance and contextual awareness, they commonly face challenges in real-time deployment, domain generalization, and resource constraints. These observations motivate our proposed multimodal, ensemble-fusion framework.

## 3. Background: Feature Representation and Architectures

This section focuses on the data preparation, feature engineering, and model architectures fundamental to the intrusion detection framework of this work. We emphasize techniques for converting NetFlow records into rich feature representations and describe two transformer-based models [32] that process these modalities.

### 3.1. Preprocessing and Feature Encoding

- Label Encoding: Categorical variables (specifically attack labels) are transformed into integer representations through label encoding. By mapping each unique category to a distinct integer, classification algorithms can interpret target classes numerically without inflating feature dimensionality through one-hot encoding.
- Text Data Conversion: To leverage language models, structured NetFlow rows are serialized into sentence-like strings, where numerical and categorical fields are concatenated into a textual sequence. These sentences are tokenized with the specific LLM’s tokenizer, then batched using a DataCollatorWithPadding for dynamic padding. This process enables transformer models to ingest tabular data as natural language, exploiting contextual embedding capabilities.
- Numeric Feature Preparation: Numeric fields are normalized via Min-Max scaling to [−1,1], center-aligning inputs for neural network stability. IP addresses are decomposed into their octets and cast to integers to preserve subnet structure. High-precision floats are downcast (e.g., float64 to float32) to reduce memory footprint without significant performance loss in large-scale IoT scenarios.

### 3.2. Advanced Feature Modalities

- Graph-Based Embeddings: We construct a similarity graph by connecting each NetFlow sample to its *k*-nearest neighbors, using FAISS for efficient neighbor search in high dimensions [33,34]. Node2Vec [35,36] then runs biased random walks on this graph, generating node sequences akin to sentences. Applying a skip-gram model yields embeddings that preserve both local homophily and global structural equivalence, producing vector representations that capture inter-sample relationships beyond raw statistics.
- Quantum-Inspired Encoding: Classical features are mapped to qubit states via AngleEmbedding [37], optionally after PCA-based dimensionality reduction. Each feature controls a Y-axis rotation on a qubit. The qubits are then measured using the Pauli-Z operator, producing real-valued outputs in [−1,1]. These values form a quantum-inspired nonlinear feature representation without requiring quantum hardware [38]. This representation is intended to provide an alternative nonlinear feature space that complements other modalities within the multimodal fusion framework.

### 3.3. Transformer-Based Architectures

- FT-Transformer: FT-Transformer stands for ’Feature Tokenizer Transformer’, an architecture that treats each numeric or categorical feature as a token and models their interactions via self-attention [39]. A feature tokenizer maps each feature to an embedding vector, and a special "[CLS]" token is prepended to aggregate the information. The sequence passes through multiple transformer blocks, where self-attention captures contextual dependencies across features. The final [CLS] embedding is used for classification, enabling the model to learn pairwise and higher-order feature relationships in an end-to-end manner.
- ELECTRA-Small: ELECTRA stands for ’Efficiently Learning an Encoder that Classifies Token Replacements Accurately’, and ELECTRA-Small is its lightweight variant optimized for constrained environments [40]. Unlike BERT’s masked-language-modeling objective [41], ELECTRA uses a generator–discriminator framework: a generator proposes token replacements, and a discriminator learns to identify which tokens are original versus replaced. Training on all input tokens, rather than just masked ones, yields faster convergence and greater sample efficiency compared to BERT. After pretraining, only the discriminator is fine-tuned for classification using the [CLS] token embedding. The model processes text converted from NetFlow rows, leveraging WordPiece tokenization [42] and dynamic padding to handle variable-length sequences.

In addition, ELECTRA-Small is a transformer-based language model (LLM) that utilizes a replaced token detection objective and self-attention to efficiently model sequential text dependencies. FT-Transformer is not an LLM; it applies the same self-attention mechanism to tabular data by tokenizing each numeric or categorical feature, eliminating the need for manual preprocessing. Both scale via transformer blocks, but they differ in pretraining design and input representation.

This background establishes how diverse feature modalities are unified and processed by advanced transformer-based models. FT-Transformer and ELECTRA-Small complement each other: FT-Transformer excels on structured tokens, while ELECTRA-Small offers efficient, fine-grained language understanding with superior training efficiency relative to BERT. The next section builds on this foundation by detailing the multimodal IDS framework and the ensemble-fusion strategies that integrate these representations.

## 4. Materials and Methods

### 4.1. System Overview

This section describes our proposed MM-NIDS framework, focusing on the overall system architecture and the primary workflow algorithm.

#### 4.1.1. System Architecture

**Figure 1:**
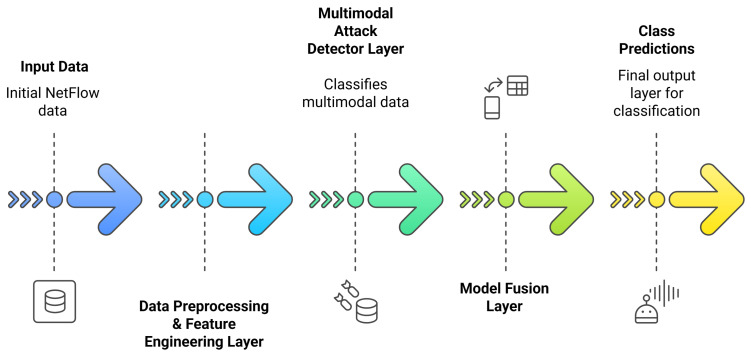
High-level architecture of the proposed MM-NIDS framework with preprocessing and feature engineering, modality-specific attack detection, and post hoc fusion layers.

**Figure 2:**
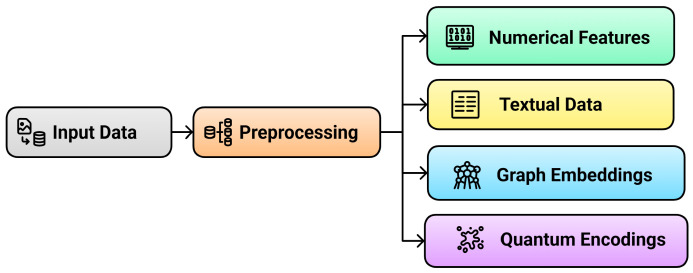
Data preprocessing and feature engineering layer.

**Figure 3:**
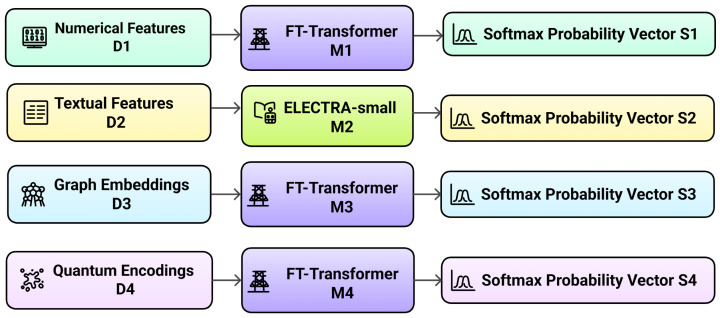
Multimodal attack detector layer.

**Figure 4:**
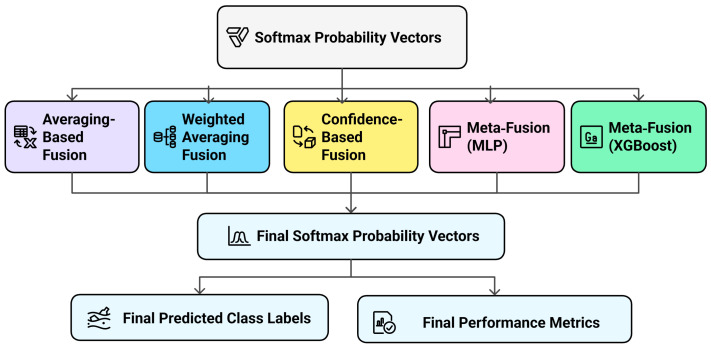
Model fusion layer using post hoc softmax-level meta-fusion.

Figure 1 presents the overall architecture of the proposed MM-NIDS. The system is organized into three modular layers:

1. Data Preprocessing and Feature Engineering Layer: Raw NetFlow records are transformed into four distinct complementary representations: structured numeric features, serialized text strings, graph-based embeddings, and quantum-inspired encodings. Figure 2 illustrates this transformation pipeline.
2. Multimodal Attack Detector Layer: Each modality is processed independently using a specialized classifier (FT-Transformer for numeric, graph, and quantum inputs, and ELECTRA-Small for text). These models operate in parallel and output softmax probability vectors. For readability in the experimental results, the four base detectors are denoted as M1 (numerical), M2 (textual), M3 (graph-based), and M4 (quantum-inspired). Figure 3 shows the four independent branches.
3. Model Fusion Layer: The softmax outputs from selected modality combinations (binary, triple, or quad) are aggregated using post hoc fusion strategies to produce the final intrusion predictions with improved accuracy and robustness. Fusion is applied after all base models are trained and evaluated, ensuring no interaction with the training pipeline. Figure 4 shows the workflow of the fusion.

#### 4.1.2. Overall Workflow

Algorithm 1 outlines our MM-NIDS pipeline. The process begins with label encoding and modality-specific feature engineering, followed by independent training and inference of the four modality classifiers. The final stage applies post hoc softmax-level fusion on the test-set probability vectors to consolidate multimodal evidence into a single intrusion prediction.
**Algorithm 1** The Proposed MM-NIDS Workflow**Input:** Raw NetFlow dataset *D***Output:** Final predicted class labels $\hat{y}$Label Encoding: Convert categorical class labels in *D* into numeric targets *y*.Preprocessing and Feature Engineering: Transform *D* into four modality-specific representations:$D_{\mathrm{numeric}}$: Structured numerical features$D_{\mathrm{text}}$: Serialized text sequences$D_{\mathrm{graph}}$: Graph embeddings via Node2Vec (Algorithm 2)$D_{\mathrm{quantum}}$: Quantum-inspired encodings via AngleEmbedding (Algorithm 3)Independent Model Training and Inference: Apply the appropriate classifier to each modality, train on the training subset, and obtain softmax probability vectors on the test set:$S_{1}\leftarrow$ FT-Transformer on $D_{\mathrm{numeric}}$$S_{2}\leftarrow$ ELECTRA-Small on $D_{\mathrm{text}}$$S_{3}\leftarrow$ FT-Transformer on $D_{\mathrm{graph}}$$S_{4}\leftarrow$ FT-Transformer on $D_{\mathrm{quantum}}$Post Hoc Fusion: Combine the softmax vectors using ensemble and meta-fusion strategies:
$\hat{y}\leftarrow$ Fusion of $\left\{S_{1},S_{2},S_{3},S_{4}\right\}$ via Algorithm 4

#### 4.1.3. Feature Engineering Layer

Graph representations capture relational patterns in traffic that raw features may miss. NetFlow data are modeled as a similarity graph, where each node corresponds to a traffic sample connected to its *k*-nearest neighbors based on feature distance. The resulting node embeddings are treated as tabular feature vectors for the FT-Transformer, allowing graph-derived structural information to be integrated with other modalities. The graph embedding generation is described in Algorithm 2.
**Algorithm 2** Generating Graph Embeddings with Node2Vec**Input:** Feature matrix $X\in R^{n\times d}$, number of neighbors *k*, embedding dimension *D***Output:** Node embeddings $E\in R^{n\times D}$Compute k-nearest neighbors for each sample using FAISSConstruct undirected graph G=(V,E) where each node is a sample, and edges link to *k* nearest neighborsInitialize Node2Vec model with walk and context parametersTrain the Node2Vec model on *G* using biased random walks**Return** node embeddings *E*

Quantum encoding is implemented as described in Section 3.2, where classical features are mapped to qubit rotations and measured via Pauli-Z to generate real-valued quantum-derived vectors. The encoding technique is summarized in Algorithm 3.
**Algorithm 3** Quantum Feature Encoding**Input:** Classical dataset $X\in R^{n\times d}$, number of qubits *Q***Output:** Quantum-encoded dataset $X_{\mathrm{quantum}}\in R^{n\times Q}$**If** Q<d **then** apply PCA to reduce *X* to $X_{\mathrm{transformed}}\in R^{n\times Q}$**Else** set $X_{\mathrm{transformed}}\leftarrow X$**For each** sample $x_{i}\in X_{\mathrm{transformed}}$ **do**(a)Initialize quantum circuit with *Q* qubits(b)Encode $x_{i}$ using AngleEmbedding (Y-axis rotations)(c)Measure each qubit using the Pauli-Z operator to get $z_{i}\in R^{Q}$**Return** $X_{\mathrm{quantum}}={\left\{z_{i}\right\}}_{i=1}^{n}$

#### 4.1.4. Transformer-Based Attack Detector Layer

This layer employs transformer topologies to learn complex intrusion patterns from multiple feature modalities. FT-Transformer processes structured numeric inputs, while ELECTRA-Small handles text-based representations, both leveraging self-attention [32] to capture contextual dependencies among features.

The FT-Transformer operates directly on normalized tabular features, embedding each dimension and modeling inter-feature relationships via stacked attention blocks. The resulting representation is passed through a classification head that outputs softmax probabilities and predicted attack classes. This design allows the model to learn both direct and higher-order dependencies among features through its attention mechanism. For the text-based modality, ELECTRA-Small is fine-tuned on tokenized sequences that describe network flow attributes in textual form. Its discriminator network encodes these sequences to classify each traffic sample as Benign or belonging to a specific attack type. Together, these transformer-based detectors provide complementary perspectives that enhance the robustness and adaptability of the overall multimodal IDS framework.

#### 4.1.5. Model Fusion Strategies

This subsection describes the post hoc fusion layer, which aggregates the softmax probability vectors produced by the four modality-specific classifiers. Fusion is applied exclusively to softmax output from the test set, ensuring that no information from the training process is reused or leaked across models. Algorithm 4 enumerates all valid combinations of modality and applies a suite of ensemble methods (averaging, weighted averaging, confidence-based fusion, and two softmax-level meta-fusion approaches) to obtain the final intrusion predictions. This modular design enables systematic comparison of fusion strategies in terms of accuracy and computational cost.
**Algorithm 4** Fusion Workflow Using Softmax-Level Post Hoc Meta-Fusion**Input:** Softmax outputs $S_{1},S_{2},S_{3},S_{4}$ from four base models (test set only); ground truth labels *y***Output:** Predictions and evaluation metrics for each fusion method and model combination; best-performing fusion setupGenerate all valid model combinations of size 2, 3, and 4 using $\left\{S_{1},S_{2},S_{3},S_{4}\right\}$For each model combination $C_{j}$:(a)Apply the following fusion methods to the softmax vectors in $C_{j}$:Averaging-Based Fusion → get predictions $P_{j,1}$, metrics $M_{j,1}$Weighted Averaging Fusion → get predictions $P_{j,2}$, metrics $M_{j,2}$Confidence-Based Fusion → get predictions $P_{j,3}$, metrics $M_{j,3}$Meta-Fusion (MLP) → get predictions $P_{j,4}$, metrics $M_{j,4}$Meta-Fusion (XGBoost) → get predictions $P_{j,5}$, metrics $M_{j,5}$(b)Record all predictions $\left\{P_{j,k}\right\}$ and metrics $\left\{M_{j,k}\right\}$ for the combination $C_{j}$Identify the best-performing fusion method and model combination.**Return** all predictions and metrics across combinations; output the best-performing setup

This fusion layer leverages the complementary strengths of the four modalities: numeric and graph embeddings capture statistical and relational structure; quantum encodings introduce nonlinear feature transformations; and textual serialization with ELECTRA provides semantic cues. Because fusion operates strictly post hoc on softmax outputs, the framework remains modular, extensible, and free from training-time interactions or data leakage.

### 4.2. Dataset Description

#### 4.2.1. Datasets

In this study, we use four NetFlow-based intrusion detection datasets: NF-BoT-IoT, NF-ToN-IoT, NF-UNSW-NB15, and NF-CSE-CIC-IDS2018 [43,44]. Following common practice in prior NetFlow-based IDS studies, all datasets were split into 80% training and 20% test sets using a stratified random row-wise partition. No explicit grouping by source or destination IP address was applied. We acknowledge that this setting may allow records involving the same hosts to appear in both training and test sets, meaning IP-derived features may capture recurring host-specific patterns rather than only general attack behavior. However, performance is not uniformly near-perfect across all datasets, and the proposed multimodal framework combines multiple feature views rather than relying solely on IP-derived numeric fields.

NF-BoT-IoT contains 600,100 total records, of which 480,080 are used for training and 120,020 for testing. NF-ToN-IoT comprises 1,379,274 records, split into 1,103,419 for training and 275,855 for testing. NF-UNSW-NB15 has 1,623,118 records, with 1,298,494 allocated to the training set and 324,624 to the test set. Finally, NF-CSE-CIC-IDS2018 is the largest, containing 8,392,401 records, of which 6,713,920 are in the training partition and 1,678,481 in the test partition.

The datasets differ substantially in both size and class composition, factors that can greatly influence model performance. One common challenge across all four datasets is severe class imbalance. The detailed breakdown of class distributions for each dataset is provided in Table 2.

In the NF-BoT-IoT dataset, attacks dominate, with only 2.31% of benign records. Reconnaissance is the largest class (78.43%), followed by DDoS and DoS (each 9.47%), while Theft (0.32%) is rare. The significant class disparity might skew models to identify classes that occur frequently. The NF-ToN-IoT dataset is more balanced. Injection accounts for 33.97% and DDoS for 23.66%, whereas benign traffic makes up 19.60%. Minority classes like Ransomware (0.01%) and MitM (0.09%) are still difficult to detect. The NF-UNSW-NB15 dataset is heavily biased towards benign traffic (95.54%), with all attacks underrepresented. Exploits (1.52%) and fuzzers (1.20%) are the most common kind of attacks, while worms (0.01%) and shellcode (0.08%) are exceedingly rare, complicating rare attack identification. The NF-CSE-CIC-IDS2018 dataset is likewise dominated by benign traffic, which comprises 87.86% of all flows. Among the attack classes, Distributed Denial of Service (DDoS) is the largest (4.53%), followed by brute-force attacks (3.48%) and DoS (3.21%). Other attack classes such as infiltration represent just 0.74%, while bot traffic contributes 0.19% of the total. Injection attacks are extremely rare (less than 0.01%), making them especially difficult to detect.

The benchmark datasets show considerable class imbalances, which can bias model performance toward majority classes. Aside from class distribution, detection performance may also depend on the feature set and how well it captures attack characteristics. The next subsections introduce the NetFlow V1 feature set and propose a hypothesis on the detectability of attacks using these features.

#### 4.2.2. NetFlow V1 Feature Set

All datasets used in this paper share the NetFlow V1 format, which includes two label fields and 12 flow-level attributes, as shown in Table 3.

These payload-free features are particularly advantageous for scalable, privacy-preserving intrusion detection, as they summarize communication behavior between endpoints. The features capture IP addresses, ports, protocols, traffic volume, and flow duration, providing both statistical and semantic context for a wide range of detection approaches—from simple heuristics to advanced machine learning algorithms. Specific fields, such as TCP flags and port numbers, help pinpoint certain attack types (e.g., floods or scans), while byte counts and flow duration serve as behavioral indicators that distinguish malicious from benign sessions. The label fields offer binary and multiclass annotations; this study utilizes only the multiclass label to identify distinct attack types during supervised classification.

### 4.3. Attack Detectability Hypothesis

NetFlow data does not capture all threats equally. Previous research on flow-based intrusion detection systems shows that flow metadata is effective for attacks such as DoS, Scanning, Worms, and Botnets, which exhibit clear changes in traffic volume or communication patterns. In contrast, payload-dependent attacks are harder to detect because flow records do not inspect packet contents [45,46].

Based on these observations, we propose the NetFlow attack detectability hypothesis as a structured evaluation lens for this study. For analysis, we classify attacks according to their visibility in NetFlow features into three detectability levels:

- High: Includes attacks such as DoS, DDoS, Scanning, Reconnaissance, and Password attacks. These tend to create clear behavioral patterns in NetFlow data, such as burst traffic, repeated access, or port scans.
- Medium: Includes Ransomware, Worms, Data Theft, Exploits, Fuzzers, and Analysis. Their visibility depends on secondary behaviors, such as propagation, exfiltration, or irregular flow timing.
- Low: Includes Injection, XSS, Backdoors, MiTM, Shellcode, and Generic attacks. These rely on payload manipulation or subtle behavior not captured in flow summaries.

This classification is a working hypothesis that will be reviewed during experimental evaluation. As illustrated in Table 4, flow-based approaches are often effective for detecting volumetric attacks (DoS, DDoS) as well as behavior-driven Reconnaissance/Scanning, since these leave visible patterns in flow metadata. In contrast, the absence of packet-level content makes it harder to identify payload-dependent vulnerabilities such as Injection, Backdoors, and man-in-the-middle (MiTM) attacks. Recognizing these distinctions is crucial for understanding the coverage limitations of NetFlow-based detection.

## 5. Experimental Results and Performance Analysis

This section describes the experimental evaluation of the proposed framework using four benchmark datasets. The evaluation focuses on the contribution of the proposed NetFlow-derived representation branches and fusion strategies under a unified experimental setting. While external IDS methods are reviewed in Section 2, our experiments emphasize multimodal representation fusion, cross-dataset behavior, and class-wise detectability. The results are organized into two primary stages:

1. Overall Performance: A high-level comparative analysis of base models and fusion strategies using aggregated metrics (accuracy, precision, recall, and F1-score).
2. Granular Detectability Analysis: A deeper investigation using confusion matrices and per-class true positive rates (TPR) to assess the model’s effectiveness against specific attack types, evaluated via our proposed ’Detection Meter’ criteria.

Our experiments use four base models, each leveraging a distinct feature modality:

- M1: Numerical-based model using core NetFlow features.
- M2: Text-based model utilizing tokenized flow-level textual representations.
- M3: Graph-based model incorporating graph-structured network flow data.
- M4: Quantum-inspired model leveraging quantum feature encodings.

M1, M3, and M4 use the FT-Transformer, a classifier suited for numeric input. M2 uses ELECTRA-Small, as it operates on text-based representations.

### 5.1. Performance Metrics

We treat intrusion detection as a multiclass classification problem involving benign traffic and specific attack types. For class-wise evaluation, let $C_{i}$ denote the class currently being evaluated, where $C_{i}$ may be the benign class or any specific attack class. The confusion-matrix outcomes are interpreted as follows:

- **True Positive (TP):** Actual class is $C_{i}$→ predicted as $C_{i}$. (Correctly identified)
- **True Negative (TN):** Actual class is not $C_{i}$→ predicted as not $C_{i}$. (Correctly not assigned to class $C_{i}$)
- **False Positive (FP):** Actual class is not $C_{i}$→ predicted as $C_{i}$. (False alarm for class $C_{i}$)
- **False Negative (FN):** Actual class is $C_{i}$→ predicted as not $C_{i}$. (Missed detection for class $C_{i}$)

From these class-wise quantities and the overall prediction outcomes, we compute four standard metrics:

1. **Accuracy:** Proportion of correctly classified samples in all classes.
  (1) $$\mathrm{Accuracy}=\frac{\mathrm{Total}\;\mathrm{correct}\;\mathrm{predictions}}{\mathrm{Total}\;\mathrm{number}\;\mathrm{of}\;\mathrm{samples}}$$
2. **Precision:** Of all samples predicted as class $C_{i}$, the fraction that truly belongs to $C_{i}$.
  (2) $${\mathrm{Precision}}_{i}=\frac{TP_{i}}{TP_{i}+FP_{i}}$$
3. **Recall:** Of all actual instances of class $C_{i}$, the fraction correctly identified.
  (3) $${\mathrm{Recall}}_{i}=\frac{TP_{i}}{TP_{i}+FN_{i}}$$
4. **F1-Score:** Harmonic mean of precision and recall for class $C_{i}$.
  (4) $${F1-\mathrm{score}}_{i}=\frac{2\cdot {\mathrm{Precision}}_{i}\cdot {\mathrm{Recall}}_{i}}{{\mathrm{Precision}}_{i}+{\mathrm{Recall}}_{i}}$$

Accuracy, precision, recall, and F1 are the commonly used global evaluation metrics in NetFlow-based intrusion detection research. These criteria guarantee that the four benchmark datasets may be directly compared with earlier IDS work. To gain a deeper understanding of class-level behavior and model performance across several attack categories, we also report confusion matrices and per-class true positive rates (TPR) in addition to these standard global measures.

### 5.2. Global Performance Evaluation: DB1 to DB4

#### 5.2.1. Results on DB1: NF-BoT-IoT Dataset

Table 5 summarizes the performance of the base models, plus the best-performing fusion results across all binary, triple, and quad model combinations on the NF-BoT-IoT dataset. Model M2 achieved the highest observed performance across all metrics, recording the highest accuracy (84.33%), precision (87.54%), recall (84.33%), and F1-score (85.71%), confirming its overall effectiveness in attack detection. M3 consistently ranked lowest, especially in precision (79.80%).

Among the ensemble configurations, the M2_M3_M4 fusion achieved the highest accuracy (85.19%) and recall (85.19%), while the M1_M2 ensemble yielded the best precision (88.13%). The top F1-score (85.46%) was obtained by the M1_M2_M4 combination. Compared to the best individual model (M2), these ensembles provided modest gains in accuracy and recall (each improving by 0.86%). Although M1_M2 showed a slight precision gain (0.59%), all other ensemble combinations underperformed in this metric, so precision improvement was excluded from the comparison plots. The best F1-score from fusion showed a small decline of 0.25% compared to M2, indicating that fusion did not enhance F1 performance in this case.

#### 5.2.2. Results on DB2: NF-ToN-IoT Dataset

Table 6 summarizes the performance of the base models, plus the best-performing fusion results in all combinations of binary, triple, and quad models on the NF-ToN-IoT dataset. Model M2 achieved the strongest performance on the NF-ToN-IoT dataset, leading across all four evaluation metrics with an accuracy of 72.57%, precision of 68.57%, recall of 72.57%, and F1-score of 68.97%, confirming its robustness in handling flow-based attack detection. In contrast, M3 consistently underperformed, particularly in F1-score (64.75%), suggesting limited generalization capacity.

For the NF-ToN-IoT dataset, the M2_M4 fusion achieved the highest accuracy, reaching 72.78%, slightly surpassing the best base model (M2) with a relative improvement of 0.21%. In terms of precision, the most notable improvement was achieved by the M2_M3_M4 fusion, which recorded a precision of 71.98%, reflecting a 3.41% gain over M2. However, no fusion model surpassed the base model M2 in F1-score, where the top-performing ensemble (M1_M2) achieved 67.91%—a 1.06% drop from M2’s 68.97%. Overall, these results in Table 6 suggest that fusion strategies offered modest gains in accuracy and recall, and one configuration notably improved precision. However, the decline in F1-score across all ensembles indicates that the balance between precision and recall was not consistently enhanced through fusion on this dataset.

#### 5.2.3. Results on DB3: NF-UNSW-NB15 Dataset

Table 7 summarizes the best-performing fusion strategies across all binary, triple, and quad model combinations on the NF-UNSW-NB15 dataset. Model M2 delivered the highest overall performance on the NF-UNSW-NB15 dataset, achieving the best accuracy (97.80%), precision (97.35%), recall (97.80%), and F1-score (97.43%), underscoring its strong generalization capability in binary intrusion detection. In contrast, M4 underperformed across all metrics, with the lowest accuracy (96.91%), recall (96.91%), and F1-score (96.43%), highlighting challenges in representing quantum features effectively in this context.

The performance variation among base models on the NF-UNSW-NB15 dataset can be attributed to the nature and dimensionality of their input features. Specifically, M1 operated on 18 numerical features, M2 utilized 12 textual features concatenated into a single sentence, M3 relied on a 32-dimensional graph embedding, and M4 processed 18-dimensional quantum encoded vectors.

For the NF-UNSW-NB15 dataset, the M1_M2_M3_M4 (quad fusion) configuration achieved the best performance across all four evaluation metrics. It recorded an accuracy of 97.88%, precision of 97.59%, recall of 97.88%, and F1-score of 97.69%. These results show consistent improvements over the best individual model, M2, with gains of 0.08% in accuracy and recall, 0.24% in precision, and 0.26% in F1-score.

Unlike other datasets where fusion sometimes led to performance drops (especially in F1-score), all metrics on the NF-UNSW-NB15 dataset benefited from ensemble learning. This highlights the complementary nature of the diverse feature representations used by M1–M4 and suggests that integrating multiple modalities leads to a more balanced and robust classifier for this specific dataset.

#### 5.2.4. Results on DB4: NF-CSE-CIC-IDS2018 Dataset

Table 8 summarizes the performance of the base models and the best-performing fusion strategies on the NF-CSE-CIC-IDS2018 dataset. Among the base models, M2 (textual features) achieved the strongest results, leading across all four evaluation metrics with an accuracy, precision, recall and F1-score above 99%. This indicates strong generalization and consistent classification performance. M3 (graph features), on the other hand, notably underperformed, particularly in accuracy (94.917%) and F1-score (94.561%). This is likely due to the computational complexity associated with graph-based representations on large-scale datasets such as NF-CSE-CIC-IDS2018. Specifically, graph similarity was computed using a 20-nearest neighbor (20-NN) approach, which may not have captured fine-grained relational patterns effectively in such a high-volume setting.

The Max_Fusion_M1_M2_M3_M4 fusion attained the highest accuracy and recall, both at 99.554%, closely followed by Max_Fusion_M1_M2_M3 and Max_Fusion_M2_M3_M4. Max_Fusion _M1 _M2 _M3 achieved the best overall F1-score (99.518%), slightly improving base M2 by 0.008 percentage points. Fusion strategies consistently offered marginal gains in precision, with all triple and quad combinations achieving 99.518% precision, compared to 99.497% for M2. Although these improvements are modest due to the already high baseline performance, the results suggest only a marginal improvement in robustness, and overall performance remains largely comparable to the best individual model. This indicates that textual features alone (M2) are already highly effective on this dataset.

#### 5.2.5. Assessment of Performance Significance

The observed improvements from fusion are generally small and should be interpreted with caution. Although large-scale evaluation over four benchmark datasets (>10 million records) produces consistent performance assessments and allows cross-dataset generalization, no formal statistical significance testing was performed. As a result, generalizations about the relative advantage of specific fusion configurations are confined to empirical trends under the current experimental context. To better quantify performance variability, future research will incorporate multiple runs and statistical testing.

#### 5.2.6. Observation on Accuracy and Recall

After evaluating the base and fusion models, we observed that accuracy and recall remained identical across runs. This is because the evaluation library uses *micro-averaging,* which aggregates all true positives (TPs), false positives (FPs), and false negatives (FNs) across classes before computing metrics such as precision, recall, and F1-score.

(5) $${\mathrm{Recall}}_{\mathrm{micro}}=\frac{\sum_{i=1}^{M}{\mathrm{TP}}_{i}}{\sum_{i=1}^{M}\left({\mathrm{TP}}_{i}+{\mathrm{FN}}_{i}\right)}$$

where *M* denotes the number of classes in the dataset being evaluated. Because each sample has exactly one true class, the numerator in Equation (5) counts all correctly predicted samples, while the denominator counts all evaluated samples and is equal to *N*, the total number of samples. Therefore, the equation simplifies to

(6) $${\mathrm{Recall}}_{\mathrm{micro}}=\frac{\mathrm{Total}\;\mathrm{correct}\;\mathrm{predictions}}{N}=\mathrm{Accuracy}$$

Thus, under micro-averaging, recall becomes mathematically equivalent to accuracy. In contrast, *macro-averaging* computes recall independently for each class and then averages the results:

(7) $${\mathrm{Recall}}_{\mathrm{macro}}=\frac{1}{M}\sum_{i=1}^{M}\frac{{\mathrm{TP}}_{i}}{{\mathrm{TP}}_{i}+{\mathrm{FN}}_{i}}$$

Macro-averaging treats all classes equally, regardless of class frequency, and is especially useful when evaluating performance on imbalanced datasets. Micro-averaging, on the other hand, gives more weight to frequent classes, making it more aligned with overall accuracy.

To gain a more detailed view of how the model performs in individual classes, we analyze the per class recall, also known as the **true positive rate (TPR)**, in the confusion matrix section. Equation (8) is used to assess the detectability of each attack type individually.

(8) $${\mathrm{TPR}}_{i}=\frac{{\mathrm{TP}}_{i}}{{\mathrm{TP}}_{i}+{\mathrm{FN}}_{i}}\times 100$$

### 5.3. Class-Wise Diagnostics and Attack Detectability Assessment

We generate confusion matrices for each base model (M1–M4) across the three datasets to better understand model behavior. These matrices reveal which attack classes are frequently misclassified by showing the distribution of accurate and inaccurate predictions.

#### 5.3.1. Hypothesis Assessment Criteria: The Detection Meter

Building on this analysis, we introduce a custom **“Detection Meter”** based on TPR statistics to assess the detection performance of each type of attack. This meter provides a structured criterion for evaluating our attack detectability hypothesis. The classification is as follows:

- **TPR** < **30%:** The attack is considered weakly detectable, suggesting a limited or ambiguous representation in NetFlow-based traffic.
- **30%** ≤ **TPR** ≤ **70%:** The attack is considered moderately detectable, reflecting partially observable or context-dependent behavior.
- **TPR** > **70%:** The attack is considered highly detectable, as it exhibits strong and consistent patterns in the NetFlow characteristics.

By using this approach, we evaluate per-class detectability for each dataset, examining which models best represent particular attack types and how model diversity affects detection robustness.

#### 5.3.2. Detectability Assessment on DB1: NF-BoT-IoT

**Figure 5:**
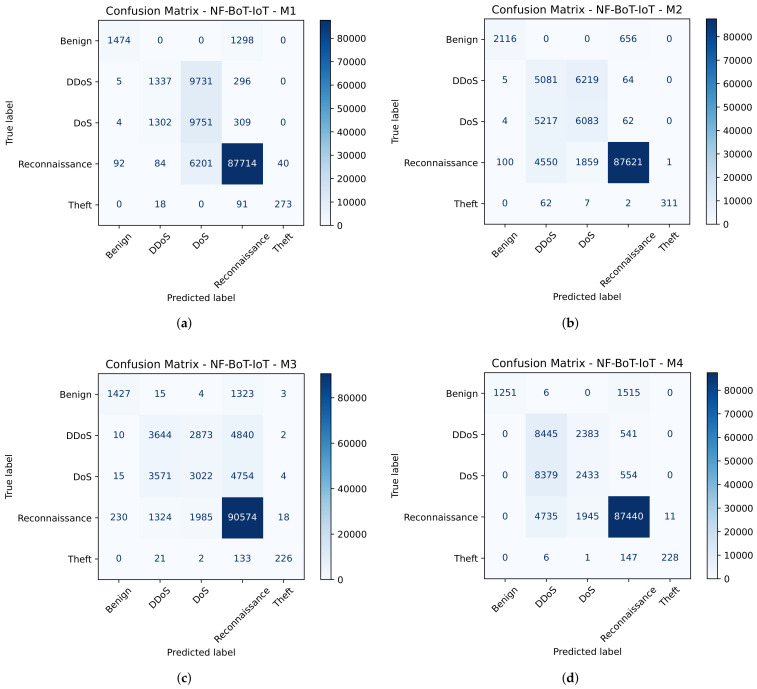
Comparison of confusion matrices across the four feature-specific base models on the NF-BoT-IoT dataset. The matrices illustrate the distribution of predicted vs. true labels for benign and attack classes: (**a**) M1; (**b**) M2; (**c**) M3; (**d**) M4. Darker blue shading indicates a higher frequency of classifications.

Figure 5a–d display the confusion matrices for the four base models on the NF-BoT-IoT dataset. These visualizations illustrate how each model performs at the class level. The NF-BoT-IoT dataset is shown here as a representative example, while the corresponding confusion matrices for the remaining datasets are provided in Appendix A. As expected, the majority of correct predictions are concentrated around high-frequency attacks such as Reconnaissance. This trend reflects the influence of class imbalance in the dataset, which biases model performance toward dominant attack types. To more precisely assess detection effectiveness across all classes, we next compute the TPR for each attack type.

Table 9 presents the evaluation of the NetFlow-based attack detectability hypothesis across four base models (M1–M4) using the BoT-IoT dataset. Each traffic class was assessed using its TPR and then compared against the detectability thresholds defined by our Detection Meter. To visually support the hypothesis, the first column is color-coded to reflect the expected detectability level of each class: high (green), medium (yellow), or low (red). We also include the relative frequency of each class in the dataset to highlight potential effects of class imbalance on detection accuracy. Beyond the raw TPR values, the table identifies the model with the highest detection rate for each class. For example, for DB1 (NF-BoT-IoT), Model M3 is the best at detecting Reconnaissance, while M4 performs best on DDoS, M1 on DoS, and M2 on Benign and Theft. These distinctions demonstrate how different feature modalities impact the models’ effectiveness.

Detectability labels (last column in the table) were determined by applying our Detection Meter to the observed TPRs. If the highest TPR for a class exceeds 70%, we classify it as highly detectable; between 30% and 70% as moderately detectable; and below 30% as weakly detectable. These derived labels are then compared with our initial hypothesis to determine whether the detection outcomes support, partially support, or contradict it. For instance, in DB1 section of Table 9, DDoS was hypothesized to be highly detectable. While Model M4 achieved a TPR of 74.28% (qualifying as highly detectable), other models fell below the threshold. Therefore, we consider this as partial support for the hypothesis.

Overall, the results for NF-BoT-IoT generally align with the hypothesis. Reconnaissance, the most dominant class, achieved high TPRs across all models, with M3 reaching 96.22%, confirming its strong NetFlow signature and high detectability. DoS and DDoS, though frequently confused with each other and with Reconnaissance, showed acceptable detection rates, supporting the hypothesis to varying degrees. The medium-detectability Theft class was best handled by M2, though misclassifications suggest its flow characteristics are not as distinct. Benign traffic, while not an attack, was often misclassified, particularly as Reconnaissance, indicating a potential sensitivity in some models to dominant attack patterns. In summary, our analysis validates the detectability hypothesis for the NF-BoT-IoT dataset, particularly for high-frequency and flow-dominant attack classes. At the same time, it highlights the benefits of leveraging diverse model architectures to capture a wider range of behaviors in NetFlow traffic.

#### 5.3.3. Detectability Assessment on DB2: NF-ToN-IoT

**Figure A1:**
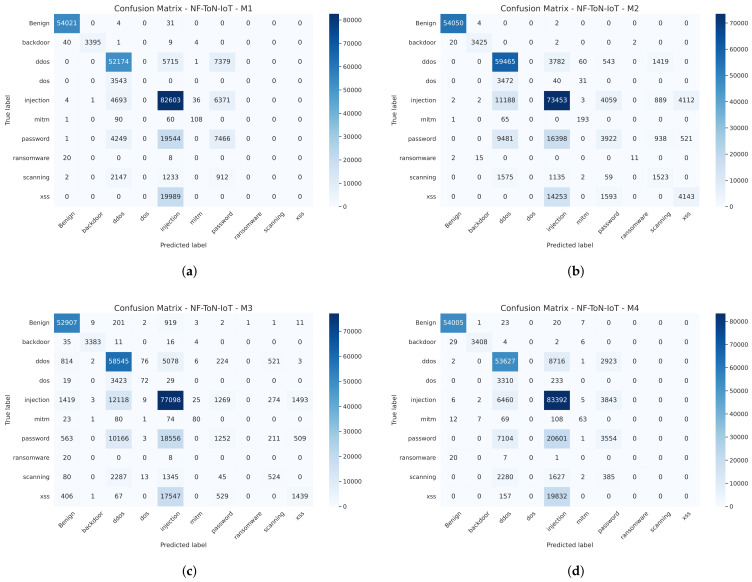
Confusion matrices for M1–M4 on the NF-ToN-IoT dataset. (**a**) M1; (**b**) M2; (**c**) M3; (**d**) M4. Darker blue shading indicates a higher frequency of classifications.

For DB2 (NF-ToN-IoT), we computed the confusion matrices for the base models to analyze class-wise prediction distributions (Figure A1). Correct predictions are concentrated around more frequent attack types, such as Injection and DDoS, while rare classes such as DoS and MiTM remain difficult to detect, consistent with the trends observed in DB1. To better understand these patterns and model limitations, we focus on a per-class TPR analysis in the discussion below.

In DB2 section of Table 9, we present the detectability results for the NF-ToN-IoT dataset across models M1–M4. As with NF-BoT-IoT, TPR values are used to evaluate each class. The first column is color-coded based on our hypothesis (high, medium, or low detectability), and class proportions are included to account for potential imbalance effects. The best-performing model for each class is also indicated. The DDoS class, expected to be highly detectable, is indeed well classified across all models, especially by M2 (91.11%), offering strong support for the hypothesis. Injection, however, was hypothesized to be weakly detectable. Contrary to expectations, it was consistently detected with relatively high TPRs, particularly by M4 (88.99%) and M1 (88.15%), thus contradicting the hypothesis. For Password, Scanning, and DoS, which were expected to be highly detectable, detection rates were mostly low. Password and Scanning attacks achieved TPRs below 40% across all models, and DoS was almost entirely missed. These results do not support the hypothesis, suggesting that class imbalance and lack of distinctive flow features limited detection. As for Backdoor and MiTM, both hypothesized to be weakly detectable, the results were mixed. Backdoor attacks were detected with high accuracy, contradicting the hypothesis. MiTM, on the other hand, achieved modest TPRs (e.g., 24.32% for M4), offering only partial support for weak detectability. Ransomware, a rare class hypothesized to have medium detectability, was inconsistently detected, with only one model (M2) reaching 39.29%, indicating partial support. Benign traffic was nearly perfectly identified by all models, confirming strong model separation from malicious flows.

In summary, the NF-ToN-IoT results show a mixed level of alignment with the detection hypothesis. Although DDoS detection clearly supports the hypothesis, the unexpected success in detecting Injection and Backdoor attacks and the poor detection of other supposedly “high-detectable” classes highlight the limitations imposed by class imbalance and overlapping NetFlow behaviors. These findings reinforce the need for complementary feature representations and tailored model strategies.

#### 5.3.4. Detectability Assessment on DB3: NF-UNSW-NB15

**Figure A2:**
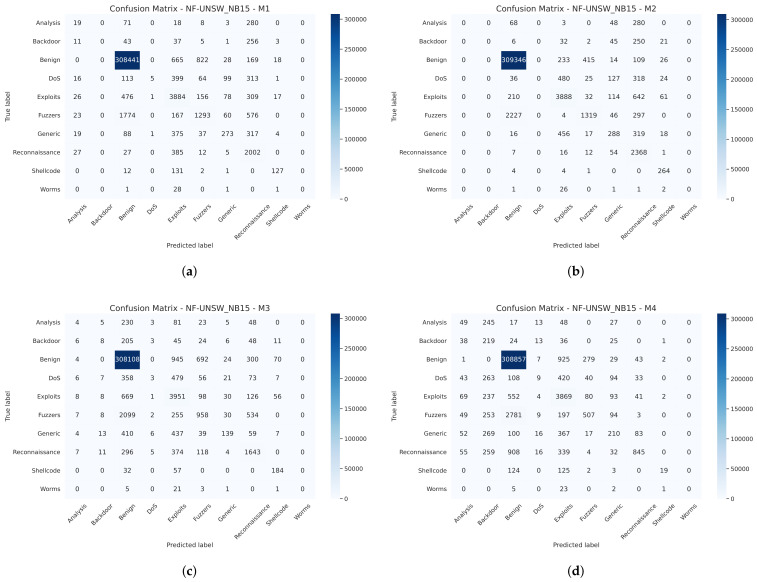
Confusion matrices for M1–M4 on the NF-UNSW-NB15 dataset. (**a**) M1; (**b**) M2; (**c**) M3; (**d**) M4. Darker blue shading indicates a higher frequency of classifications.

As with the previous datasets, confusion matrices were computed for the base models to examine class-wise prediction behavior on the NF-UNSW-NB15 dataset (Figure A2). All models showed strong performance in identifying benign traffic, with over 95% of benign instances correctly classified. However, detection of low-frequency attack classes (such as Backdoor, Worms, and Analysis) tends to be limited or inconsistent between models. To evaluate individual class-level performance, we examine their TPRs.

In DB3 section of Table 9, we summarize the attack detectability analysis for the NF-UNSW-NB15 dataset across models M1–M4. The first column is color-coded based on our hypothesis, and class percentages are included to contextualize detection difficulty due to imbalance. As before, the best-performing model is indicated for each class based on the TPR values.

Benign traffic, which comprises the majority of the dataset, was classified with high accuracy by all models. Reconnaissance, hypothesized to be highly detectable, achieved strong TPRs from M1 (81.45%) and M2 (96.34%), but lower performance from M3 (66.84%) and M4 (34.38%). As such, we consider this case as only partially supporting the hypothesis. In contrast, DoS, also expected to be highly detectable, was almost entirely missed across all models, with TPRs below 1%, contradicting the hypothesis.

Given the hypothesized medium detectability classes, Exploits achieved consistent TPRs around 78–80% across models, partially supporting the hypothesis by exceeding the threshold in some models. Fuzzers showed mixed results, with modest detection performance in M1 and M2, offering partial support. In contrast, Analysis remained largely undetected across all models, contradicting the hypothesis and indicating that it may behave more like a weakly detectable class. Worms were not detected at all, also failing to meet the expected medium detectability level.

For the hypothesized low-detectability group, Generic was poorly detected across all models, which supports the hypothesis. Backdoor, although expected to be weakly detectable, achieved relatively high TPR in M4. This results in partial support for the hypothesis. Shellcode, on the other hand, was strongly detected by M2 (96.70%), moderately detected by M1 and M3, and weakly detected by M4, so we consider this as partial support for the low-detectability hypothesis.

In summary, the NF-UNSW-NB15 results offer partial validation of the detectability hypothesis. While some classes behaved as expected, others (especially DoS and low-frequency attacks) challenged our assumptions. These results highlight how difficult it is to model complicated or unbalanced behaviors with just NetFlow attributes.

#### 5.3.5. Detectability Assessment on DB4: NF-CSE-CIC-IDS2018

**Figure A3:**
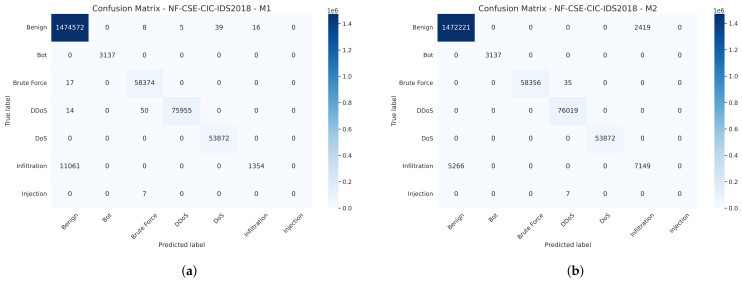

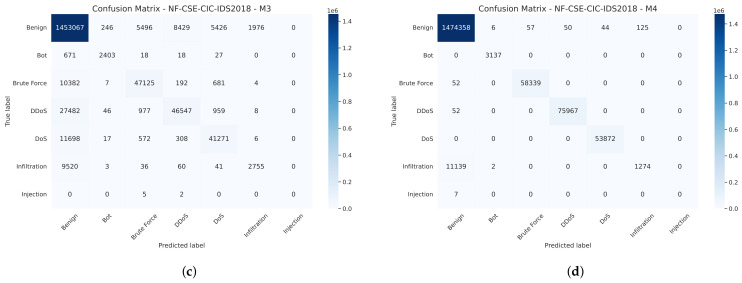
Confusion matrices for M1–M4 on the NF-CSE-CIC-IDS2018 dataset. (**a**) M1; (**b**) M2; (**c**) M3; (**d**) M4. Darker blue shading indicates a higher frequency of classifications.

For DB4 (NF-CSE-CIC-IDS2018), the confusion matrices show stronger diagonal patterns for dominant classes such as Benign, Brute Force, DDoS, and DoS (Figure A3). However, minority classes remain unevenly detected. Infiltration is frequently confused with Benign traffic, and the rare Injection class is missed across the base models. These patterns suggest that high-volume attacks are more distinguishable in flow-level features, while sparse or subtle attack classes require closer TPR-based analysis.

In DB4 section of Table 9, we summarize the attack detectability analysis for the NF-CSE-CIC-IDS2018 dataset. The first column is color-coded based on our hypothesis, and class percentages are included. As before, the best-performing model is indicated for each class based on the TPR values.

In DB4, Benign flows dominate (87.86%) and are almost perfectly identified by all models (best TPR is 99.995% by M1). Since Benign traffic is not an attack class, detectability is assessed only for the attack categories. Among attacks, DDoS (4.53%) and Brute Force (3.48%) are highly detectable—M2 achieves 100% TPR on DDoS and M1 reaches 99.971% on Brute Force, supporting the high-detectability hypothesis. DoS (3.21%) is also perfectly detected (100% TPR by M1, M2, M4). Infiltration (0.74% of DB4) yields only moderate detection by M2 with 57. 58% TPR, providing partial support for the hypothesis of medium detection. Meanwhile, Bot traffic (0.19%) is fully detected (100% TPR by M1, M2, M4), contrary to our expectation of medium detection. Finally, Injection (<0.01%) remains undetected (0% TPR), confirming its hypothesized low detectability.

In summary, the NF-CSE-CIC-IDS2018 results provide strong validation for our detectability hypothesis in volumetric attacks—DDoS, Brute Force, and DoS all achieved near-perfect TPRs. Infiltration shows only partial support; Bot traffic proved more detectable than expected and Injection remained entirely undetected, confirming its low-detectability status. These mixed outcomes underscore the limitations of NetFlow V1 features alone for modeling rare or behaviorally subtle threats and point to the need for tailored detection strategies.

#### 5.3.6. Cross-Dataset Summary of Attack Detectability Results

The general results of the evaluated datasets indicate that the proposed detectability hypothesis is *partially valid*. Table 10 summarizes the hypothesis support status for each attack class, with cell colors reflecting the hypothesized detectability level (green for high, yellow for medium, red for low), and symbols indicating whether the hypothesis was supported (**✓**) or not (**✗**).

Some attack types, such as Reconnaissance, DDoS, and Exploits, were generally well detected, confirming the hypothesis in most cases. Others, like DoS and Password, exhibited mixed results because of frequent confusion and variability between models and datasets. Meanwhile, low-detectability classes such as XSS, MiTM, and Shellcode were mostly difficult to detect, matching expectations and reinforcing the limitations of NetFlow features for stealthy or payload-based attacks.

Across all datasets, class imbalance was a recurring issue: rare attacks were often misclassified, even when their detectability was hypothesized to be moderate or high. Lastly, the diverse strengths of different models across attack types show that the combination of multiple modalities enhances the coverage and robustness of detection. Each model contributes differently, reinforcing the benefit of diverse data representations. For instance, based on Table 9:

- M1 (numerical) achieved the strongest TPR for DoS attacks in DB1 (85.79%), highlighting its strength in the capture of structured tabular traffic patterns;
- M2 (textual) performed best for Reconnaissance detection in DB3 (96.34%), showing its advantage in modeling sequential flow-based characteristics;
- M3 (graph-based) proved effective in relational settings, such as the detection of Reconnaissance in DB1 (96.22%) and Exploits in DB3 (79.87%), underscoring the importance of capturing structural dependencies between entities;
- M4 (quantum-inspired encoding) contributed complementary improvements in challenging classes, including Injection in DB2 (88.99%), and Backdoor in DB3 (61.52%), demonstrating its role in addressing under-detected attacks.

Collectively, these examples justify the inclusion of all four modalities and highlight the benefit of a multimodal IDS design.

### 5.4. Sensitivity Analysis and Hyperparameter Tuning

We conducted sensitivity tests on relevant hyperparameters and input configurations to assess the model’s resilience. The objective was to track the effects of these modifications on detection measures, including F1-score, accuracy, precision, and recall. We varied model parameters, including learning rate, training epochs, batch size, and the number of transformer layers. We also experimented with optimizing the data representations by varying the number of neighbors *k* in Node2Vec graphs, and quantum encoding dimensions. The *k* value and quantum dimensions directly shape feature quality in the graph-based (M3) and quantum-inspired (M4) models. These experiments help identify optimal settings and demonstrate the sensitivity of each model to parameter changes.

#### 5.4.1. Effect of Number of Transformer Layers

We investigated various transformer layer depths to determine their impact on classification performance. The model can learn more intricate hierarchical representations by adding layers, which could increase its accuracy when dealing with intricate patterns. However, if deeper models are not correctly regularized, they can induce overfitting or longer training times.

Based on the results in Table 11, classification using FT-Transformer with varying transformer layer depths reveals that increasing the number of layers does not necessarily lead to consistent performance improvements. Accuracy was not significantly affected by the number of layers, remaining around 83% across all configurations. The 10-layer model achieved the highest F1-score (84.91%) and precision (87.50%), while both the six-layer and 12-layer models reached the top recall. However, the 12-layer configuration also had the lowest F1-score (79.68%), indicating a drop in precision. Overall, the 10-layer model offered the best balance across all evaluation metrics.

#### 5.4.2. Effect of Learning Rate

We conducted experiments to evaluate the impact of varying learning rates on model performance. The learning rate is a key hyperparameter that controls how frequently the model’s weights are updated during training. Selecting an appropriate value is critical for stable convergence, helping avoid unstable updates or convergence to suboptimal solutions. To isolate the influence of the learning rate, all other model parameters were kept fixed while only the learning rate was varied. Given the size of the dataset, training for the learning-rate experiment was restricted to two epochs to limit computational cost.

The results in Table 12 show a clear performance trend across varying learning rates. As the learning rate increased from $1\times 10^{-5}$ to $4\times 10^{-5}$, the model mostly exhibited consistent improvements in accuracy, precision, recall, and F1-score. The model trained with a learning rate of $4\times 10^{-5}$ achieved the best overall performance, suggesting that a moderately higher learning rate enabled more effective weight updates without destabilizing training. Notably, precision showed the most significant increase, indicating an improvement in the model’s confidence in its predictions. These results underscore the importance of tuning the learning rate, as even small adjustments can have a substantial impact on classification performance, particularly when fine-tuning pretrained language models like ELECTRA-Small.

#### 5.4.3. Effect of Batch Size

We varied the training batch size to assess its impact on the classification performance. While larger batch sizes enable faster training, smaller batch sizes can offer better generalization by introducing more noise into the gradient estimation process. This experiment helps identify the optimal batch size for our task. The results in Table 13 reveal the nuanced impact of batch size on model performance. As batch size increased from 128 to 2048, the evaluation metrics exhibited minor fluctuations, with the most notable improvements observed in F1-score and accuracy. A batch size of 512 achieved the highest F1-score of 84.10%, suggesting an optimal balance between gradient noise and stability. Interestingly, although the largest batch size (2048) yielded the highest accuracy at 84.04%, it did not outperform mid-range batch sizes in terms of F1-score or precision. This suggests that while larger batches may facilitate slightly better overall accuracy, they can reduce the model’s ability to generalize across all classes. These results highlight the importance of adjusting the batch size to balance prediction accuracy and training efficiency.

#### 5.4.4. Effect of Number of Training Epochs

We evaluated the performance of the models across various training epochs to examine the relationship between training duration and classification accuracy. This experiment helps determine whether the models benefit from additional training or are prone to overfitting. Based on the results in Table 14, model performance improved consistently with an increase in training epochs. The accuracy rose from 82.39% at one epoch to 84.17% at five epochs, with corresponding gains in precision, recall, and F1-score. Notably, F1-score increased from 81.47% to 85.10%, indicating better overall balance between precision and recall as training progressed. These results suggest that the model benefits from additional training without signs of overfitting up to five epochs. However, the rate of improvement slightly diminishes beyond three epochs, which may help guide the choice of training duration for efficiency without compromising performance.

#### 5.4.5. Effect of *k* in Graph Embedding (Node2Vec)

In this experiment, we varied the number of nearest neighbors (*k*) used to build the graph structure for Node2Vec embeddings in the M3 model. The goal is to understand how local connectivity influences feature quality and downstream classification.

Table 15 shows the effect of adjusting the number of nearest neighbors (*k*) in Node2Vec graph building on the NF-ToN-IoT dataset. With k=100 obtaining the greatest values for accuracy (71.05%), precision (66.21%), recall (71.05%), and F1-score (65.55%), performance steadily improved as *k* grew. The complex and diverse flow patterns in NF-ToN-IoT are likely the reason for this tendency, which suggests that tighter network connectivity produces more useful embeddings. However, when considering the higher processing cost, the slight gain between k=50 and k=100 suggests declining returns. Therefore, k=50 might provide a useful trade-off between efficiency and performance.

#### 5.4.6. Effect of Quantum Encoding Dimensions

We evaluated how different quantum feature sizes (i.e., the number of qubits) affect the performance of the M4 model. This directly impacts the resolution of quantum-encoded representations used for multiclass classification. According to the findings in Table 16, performance on the NF-UNSW-NB15 dataset is generally improved by adding more qubits across all assessment parameters. While 18 qubits somewhat increased precision and F1-score, 16 qubits produced the best overall accuracy and recall. According to these results, feature expressiveness can be enhanced by larger quantum encoding dimensions, but improvements eventually plateau. For this dataset, 16–18 qubits provide a good trade-off between model complexity and performance.

#### 5.4.7. Key Findings from Sensitivity Analysis

The optimization study demonstrated the importance of fine-tuning input configurations and hyperparameters in improving performance across the various models and datasets analyzed. Each experiment was carefully designed to isolate a single variable, enabling a more detailed understanding of its impact on classification measures, including accuracy, precision, recall, and F1-score. The key findings for each hyperparameter are summarized below, based on experiments conducted across specific models and datasets:

- On NF-BoT-IoT using ELECTRA-Small, raising the learning rate to $4\times 10^{-5}$ yielded the highest F1-score of 83.87%, showing clear gains with moderate increases.
- For the same model and dataset, increasing epochs from one to five improved F1-score from 81.47% to 85.10%, with no signs of overfitting.
- On NF-BoT-IoT with FT-Transformer, a batch size of 512 produced the best F1-score (84.10%). Larger batches (e.g., 2048) slightly improved accuracy but reduced precision and F1.
- Varying transformer layers on FT-Transformer showed 10 layers as optimal, achieving the highest F1-score (84.91%) and precision (87.50%). The 12-layer variant had slightly better accuracy but suffered in F1 due to low precision.
- For the M3 model on NF-ToN-IoT, performance improved with increasing Node2Vec *k*, peaking at k=100 (F1: 65.55%, Acc: 71.05%), though benefits flattened beyond k=50.
- In the M4 quantum model on NF-UNSW-NB15, increasing qubits enhanced performance. Best accuracy (96.92%) was at 16 qubits; best F1-score (96.43%) and precision were at 18. Gains stabilized beyond 16 qubits.

Overall, our findings emphasize the importance of tailoring hyperparameter selection to both the model architecture and the specific dataset. The ideal configurations varied depending on the situation, although broad tendencies, such as improved performance with longer training epochs or relatively high learning rates, were observed. The quantum encoding experiments, for instance, showed that the optimal number of qubits varied depending on the dataset, indicating that what is effective for one task might not be the same for another. Therefore, rather than serving as general guidelines, these experiments are meant to serve as illustrative case studies. To obtain reliable and effective cyber threat detection, it is crucial to do model-specific and data-specific optimization.

## 6. Discussion, Conclusions and Future Work

In this work, we proposed, developed, and evaluated MM-NIDS, a novel multimodal fusion framework for NetFlow-based intrusion detection. MM-NIDS integrates four complementary NetFlow-derived representations: numerical (M1), textual (M2), graph-based (M3), and quantum-inspired (M4). The FT-Transformer architecture was used for the numerical, graph-based, and quantum-inspired branches, while ELECTRA-Small was used for the text-based branch (M2).

The FT-Transformer was chosen for its proven performance on tabular and heterogeneous data, its versatility in handling numerous modalities, and its attention-based mechanism, which enables contextual learning across diverse features—ideal for dynamic network flows.

The text-based model (M2) utilized the ELECTRA-Small transformer, selected for its efficient design and suitability for classification tasks in constrained environments. ELECTRA’s unique pretraining strategy, based on replaced token detection, enables it to learn high-quality representations more efficiently than traditional approaches. The "small" variant is ideal for intrusion detection scenarios where runtime efficiency and performance are crucial since it provides a good balance between accuracy and computational cost.

The graph-based model was incorporated to capture the topological relationships between hosts and flows. Graph embeddings reveal structural patterns in the network, such as hubs, communities, or peer-to-peer clusters, that are often indicative of scanning, flooding, or lateral movement. These behaviors are difficult to isolate using flat feature vectors, making a graph-based approach valuable for detecting such complex interactions.

The quantum-inspired encoding was used to explore whether encoding NetFlow features into quantum states could enhance representation power for subtle traffic behaviors. In our framework, each qubit represents a single normalized NetFlow feature, encoded as a rotation angle using RY gates (i.e., angle embedding). After this transformation, a measurement is performed using the Pauli-Z observable, effectively projecting the qubit state onto the Z-axis of the Bloch sphere. This approach offers a nonlinear transformation of classical features into a new space, which may aid in separating complex or overlapping attack patterns.

Fusion was used to integrate the complementary qualities of various modalities. We implemented multiple fusion strategies, including weighted averaging, confidence-based fusion, and meta-fusion using MLP and XGBoost, to combine diverse perspectives. The fusion layer integrates multiple perspectives to enhance detection resilience, as no single representation can fully capture all aspects of an attack. This enabled more robust detection, particularly for ambiguous or borderline cases, where a single view was insufficient.

Alongside model development, we presented and tested an attack detectability hypothesis, arguing that certain types of attacks exhibit greater behavioral traces in NetFlow summaries and thus are more easily traceable, while others, especially stealthy or payload-based attacks, remain elusive. This hypothesis provided interpretive value for understanding model strengths, guiding both evaluation and architectural design. We conducted a cross-dataset assessment using four publicly available NetFlow-based benchmarks: NF-BoT-IoT, NF-ToN-IoT, NF-UNSW-NB15, and NF-CSE-CIC-IDS2018. These datasets vary in terms of attack types, traffic volume, and class distribution, providing a comprehensive testbed for assessing model generalizability. We confirmed the resilience and adaptability of the proposed system in various multiclass intrusion detection settings by showcasing consistent performance across the datasets.

### 6.1. Insights from Evaluation

The evaluation results across all four datasets demonstrated several consistent trends. Most notably, the text-based model (M2), which used the ELECTRA-Small LLM, consistently delivered the strongest individual detection accuracy. This supports the effectiveness of transformer-based architectures, particularly for semantically enriched NetFlow representations.

Model fusion further improved performance in many cases. Aggregating outputs from diverse modalities allowed the ensemble models to leverage the unique strengths of each base model. While fusion did not always outperform the best individual model in all metrics, it often led to more balanced detection across attack classes, especially in complex or imbalanced datasets.

Another important finding is that increased model diversity improved the detection of underrepresented attack classes. This aligns with the broader goal of robust intrusion detection, especially when facing evolving threats. Finally, the detectability analysis confirmed that NetFlow-based features are particularly effective for volumetric and scan-based attacks, but less so for stealthy or payload-driven threats, such as Shellcode or XSS. This highlights an inherent limitation of flow-level representations and suggests the need to incorporate deeper semantic or payload-level features in future work.

### 6.2. Limitations

This study was conducted offline using preprocessed benchmark datasets, which limits its applicability to real-time deployment scenarios. The system was not evaluated under streaming or live traffic conditions, so constraints such as inference latency, throughput, and response time were not addressed.

While macro-averaged metrics were used to mitigate the effects of class imbalance, all datasets still exhibited significant skew in class distributions. This hampered the detection of unusual attack types and may have increased bias towards dominant classes, particularly in the absence of oversampling procedures.

Another limitation is the absence of cross-validation and statistical significance testing. Performance was evaluated using stratified fixed splits to preserve class balance and reduce bias, but fixed splits may still limit generalizability. While incorporating k-fold validation and hypothesis testing would further strengthen empirical robustness, these approaches are computationally demanding in practice, given the scale of the IoT datasets used in this study. We therefore acknowledge this as an open direction for future work aimed at enhancing the statistical reliability of multimodal IDS evaluation.

Graph-based and quantum-encoded models introduced higher computational overhead compared to other representations. In particular, graph construction and qubit encoding required additional memory and time during the preprocessing stage. This added to the system’s overall complexity and may pose challenges for large-scale or latency-sensitive deployments.

Finally, although quantum-inspired encoding was explored in this work, its application remains preliminary. Research is still needed to assess the consistency and additional value of quantum transformations compared to traditional feature engineering techniques.

### 6.3. Future Work

While the proposed framework shows promising results, several opportunities remain for future extension and enhancement.

- Real-time deployment: Adapt and optimize the system for live-stream processing in operational network environments, accounting for end-to-end inference latency and throughput.
- Explainable AI: Integrate interpretability techniques such as SHAP values, attention heatmaps, or rule-based logic to explain model decisions and increase trust in high-stakes settings [47].
- Improved handling of imbalance: Investigate advanced methods for addressing class imbalance, including cost-sensitive loss functions, adversarial data augmentation, and targeted oversampling for underrepresented attack classes [48].
- Refining quantum models: Enhance the standalone performance of M4 by exploring more advanced quantum encoding and training strategies [49]. For example, amplitude encoding can compactly map entire feature vectors into the amplitudes of a single quantum state, reducing qubit requirements. Other approaches, such as hybrid entangled circuits or parameterized quantum circuits, may offer richer representations and improve learning capacity.
- Adaptive fusion: Extend the current static fusion strategies to dynamic or attention-based fusion mechanisms that learn to weight model contributions based on input context.
- Cross-dataset generalization: Validate the framework on broader public datasets and real-world traffic to better assess generalizability and deployment readiness.
- Model validation and significance testing: Incorporate repeated cross-validation, confidence intervals, and statistical testing (e.g., paired *t*-tests or bootstrapping) to assess the consistency and significance of observed performance differences. Future work will assess potential identity leakage by excluding IP-derived features and using host-grouped train/test splits where possible.
- Exploratory diagnostic analyses: Future research could use diagnostic techniques, such as inter-model error correlation or simple disagreement measures, to better understand how different representations impact traffic classifications. Despite being uncommon in NetFlow-based IDS research, these studies might provide more information about complementary decision patterns and class-specific behaviors.
- Deployment efficiency: Future work will also include a detailed computational complexity analysis, including inference time, memory footprint, model size, and comparison with lightweight baselines, to assess suitability for resource-constrained deployment.

Overall, this study contributes to the development of intelligent, robust, and scalable intrusion detection systems by presenting a modular framework that can adapt to advances in network telemetry and artificial intelligence, while demonstrating the value of multimodal learning for intrusion detection.

## Figures and Tables

**Table 1:** Comparison of IDS techniques.

Ref. & Method	Dataset	Limitation
[7] Stacking ensemble, GAN-based sampling	NSL-KDD	Computational complexity
[8] BAT-MC (BiLSTM + attention)	NSL-KDD	High false negatives
[9] Hybrid LGBM-kMeans	NSL-KDD	High training time
[10] Autoencoder	NSL-KDD	Deeper models did not outperform 3-layer AE
[11] GAN-RF	CICIDS2017	Ineffective on bot/infiltration attacks
[12] GAN-ECNN	NSL-KDD, UNSW-NB15	Computational complexity
[14] GAN-One-Class	ISCX-2012, CIC-2017, CSE-2018	Lower accuracy than related methods
[13] Bi-GAN	NSL-KDD, CIC-DDoS-2019	High false positive rate on NSL-KDD
[15] k-NN, RF, SVM	NSL-KDD	Feature set and split details omitted
[16] IoV-BERT-IDS	CICIDS, BoT-IoT, Car-Hacking, IVN-IDS	Struggles with similar attacks; real-time scalability issues
[17] Transformer-based NIDS	CIC-IDS2018	High resource usage; weaker on some attacks
[18] PLLM-CS	UNSW-NB15, TON-IoT	Lack of domain-specific datasets; compute-intensive
[19] Improved transformer	NSL-KDD, UNSW-NB15	Limited generalizability; high cost
[20] TabTransformer	Military network dataset	Computational complexity
[21] Lightweight transformer-based IDS	NF-BoT-IoT, NF-ToN-IoT	Limited generalizability; limited scalability in large-scale networks
[22] BERT-GPT-LSTM	CICIoT2023	Limited coverage; misclassification issues
[23] BERT-based IDS	NSL-KDD	Computational overhead; limited validation
[24] Llama2 DDoS detector	CIC-IDS2017, CIC-DDoS2019	Complex preprocessing; limited formats
[25] LLM distillation + CNN	UNSW-NB15	Offline preprocessing limits real-time use
[26] Federated BERT NIDS	Edge-IIoTset	High preprocessing complexity
[27] LLMs (prompt, RAG, fine-tune)	Proprietary dataset	Intensive compute; poor zero-day generalization
[28] HuntGPT (RF + XAI + LLM)	KDD99	Limited real-world validation; scalability concerns
[29] ShieldGPT	CIC-DoS2017, CIC-DDoS2019	Manual validation required; needs broader testing
[30] KG + ML NIDS	KDD’99, NSL-KDD, logs, CTI	Computationally intensive; data quality-dependent
[31] ASGAFGNN (ViT+BiLSTM+GNN+FL)	CICIDS2017, UNR-IDD, NF-UQ-NIDS-v2, NSL-KDD	High preprocessing cost; dataset dependency

**Table 2:** Class distributions for the four NetFlow-based datasets.

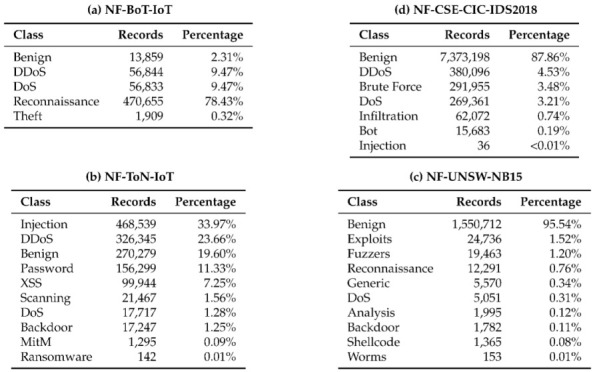

**Table 3:** NetFlow V1 features used in this study.

Feature	Description and Relevance
IPV4_SRC_ADDR	Source IP address. Helps detect scanning or compromised hosts.
IPV4_DST_ADDR	Destination IP address. Useful for identifying targets or periodic communication with attacker servers.
L4_SRC_PORT	Source port. Repeated use may indicate malware activity or evasion tactics.
L4_DST_PORT	Destination port. Key for detecting scans, brute-force attempts, or backdoor access.
PROTOCOL	Transport protocol (e.g., TCP, UDP). Helps classify attack types such as SYN floods or port scanning.
TCP_FLAGS	TCP control flags. Useful for detecting SYN floods, null scans, and other malicious behaviors.
L7_PROTO	Application-layer protocol identifier. Indicates tunneling or misuse of higher-layer protocols.
IN_BYTES	Number of incoming bytes. High volume may signal flooding; very low values may indicate scanning.
OUT_BYTES	Number of outgoing bytes. Helps detect data exfiltration or abnormal outbound traffic.
IN_PKTS	Incoming packet count. High packets with low bytes may suggest probing activity.
OUT_PKTS	Outgoing packet count. Excessive values may indicate brute-force or denial-of-service (DoS) attacks.
FLOW_DURATION_MILLISECONDS	Flow duration in milliseconds. Short flows often indicate scans; long flows may suggest tunneling or data exfiltration.
Label	Binary label (benign/attack). Used as the target for classification.
Attack	Multiclass label identifying specific attack types (e.g., DoS, Bot, Injection).

**Table 4:** NetFlow detectability of attack types.

Attack Type	Detectability *	Reasoning
DoS	High	High volume and short-duration repetitive flows.
DDoS	High	Coordinated flooding creates strong, repeated flow patterns.
Scanning	High	Short flows to many IPs or ports; easily distinguishable.
Reconnaissance	High	Similar to scanning; reveals network structure through flow behavior.
Password Attack	High	Repeated connection attempts to authentication services create repetitive flow patterns.
Brute Force	High	Repeated connection attempts create a dense, repetitive flow pattern.
Ransomware	Medium	May produce suspicious post-infection network traffic, such as unusual outbound flows.
Worms	Medium	Can resemble scanning, but spread patterns vary over time.
Data Theft	Medium	Large outbound flows may occur; timing can be inconsistent.
Exploits	Medium	Timing or port-usage changes possible; more subtle than volumetric.
Fuzzers	Medium	Irregular input may create inconsistent flow behavior.
Analysis	Medium	Indirect indicators; anomalies show up only via correlation.
Infiltration	Medium	Often mimics valid application-level flows.
Bot	Medium	Periodic check-in traffic to the attacker’s server.
Injection	Low	Payload-based; does not visibly alter flow structure.
XSS	Low	Application-layer threat; no clear NetFlow signature.
Backdoor	Low	Mimics normal traffic; flows appear typical.
MiTM	Low	Requires deep-packet inspection beyond flow metadata.
Shellcode	Low	Low-level code execution; flows often look benign.
Generic	Low	No visible anomaly in flow; affects internal logic.

* *Detectability indicates expected visibility from NetFlow-level features: High, Medium, Low.*

**Table 5:** Performance of base models and fusion strategies on the NF-BoT-IoT dataset.

Model Name	Accuracy (%)	Precision (%)	Recall (%)	F1-Score (%)
M1 (Numerical features)	83.78	87.34	83.78	83.44
M2 (Textual features)	84.33	87.54	84.33	**85.71**
M3 (Graph features)	82.40	79.80	82.40	80.72
M4 (Quantum features)	83.15	85.76	83.15	83.48
Max_Fusion_M1_M2	84.49	**88.13**	84.49	84.34
Max_Fusion_M1_M3	84.80	84.40	84.80	84.48
Max_Fusion_M1_M4	84.43	86.28	84.43	84.72
Max_Fusion_M2_M3	85.15	84.65	85.15	84.81
Max_Fusion_M2_M4	84.37	87.11	84.37	85.16
Max_Fusion_M3_M4	84.56	84.88	84.56	84.65
Max_Fusion_M1_M2_M3	85.11	85.62	85.11	84.76
Max_Fusion_M1_M2_M4	84.44	87.34	84.44	**85.46**
Max_Fusion_M1_M3_M4	84.99	84.59	84.99	84.67
Max_Fusion_M2_M3_M4	**85.19**	85.21	**85.19**	84.93
Max_Fusion_M1_M2_M3_M4	85.11	85.43	85.11	84.90

**Table 6:** Performance of models and fusion strategies on the NF-ToN-IoT dataset.

Model Name	Accuracy (%)	Precision (%)	Recall (%)	F1-Score (%)
M1 (Numerical features)	72.42	64.88	72.42	67.89
M2 (Textual features)	72.57	68.57	72.57	**68.97**
M3 (Graph features)	70.80	65.72	70.80	64.75
M4 (Quantum features)	71.79	63.08	71.79	65.94
Max_Fusion_M1_M2	72.67	68.33	72.67	**67.91**
Max_Fusion_M1_M3	72.54	67.73	72.54	67.05
Max_Fusion_M1_M4	72.74	67.41	72.74	67.68
Max_Fusion_M2_M3	72.64	67.81	72.64	67.54
Max_Fusion_M2_M4	**72.78**	68.23	**72.78**	67.67
Max_Fusion_M3_M4	72.20	69.43	72.20	66.22
Max_Fusion_M1_M2_M3	72.73	68.02	72.73	67.65
Max_Fusion_M1_M2_M4	72.71	68.34	72.71	67.88
Max_Fusion_M1_M3_M4	72.52	67.99	72.52	67.08
Max_Fusion_M2_M3_M4	72.75	**71.80**	72.75	67.61
Max_Fusion_M1_M2_M3_M4	72.72	67.89	72.72	67.65

**Table 7:** Performance of models and fusion strategies on the NF-UNSW-NB15 dataset.

Model Name	Accuracy (%)	Precision (%)	Recall (%)	F1-Score (%)
M1 (Numerical features)	97.36	97.19	97.36	97.07
M2 (Textual features)	97.80	97.35	97.80	97.43
M3 (Graph features)	97.03	96.45	97.03	96.59
M4 (Quantum features)	96.91	96.57	96.91	96.43
Max_Fusion_M1_M2	97.84	97.57	97.84	97.66
Max_Fusion_M1_M3	97.58	97.27	97.58	97.33
Max_Fusion_M1_M4	97.57	97.29	97.57	97.35
Max_Fusion_M2_M3	97.82	97.49	97.82	97.66
Max_Fusion_M2_M4	97.86	97.56	97.86	97.66
Max_Fusion_M3_M4	97.50	97.16	97.50	97.25
Max_Fusion_M1_M2_M3	97.86	97.55	97.86	97.66
Max_Fusion_M1_M2_M4	97.85	97.59	97.85	97.62
Max_Fusion_M1_M3_M4	97.58	97.23	97.58	97.34
Max_Fusion_M2_M3_M4	97.85	97.51	97.85	97.63
**Max_Fusion_M1_M2_M3_M4**	**97.88**	**97.59**	**97.88**	**97.69**

**Table 8:** Performance of models and fusion strategies on the NF-CSE-CIC-IDS2018 dataset.

Model Name	Accuracy (%)	Precision (%)	Recall (%)	F1-Score (%)
M1 (Numerical features)	99.332	99.329	99.332	99.068
M2 (Textual features)	99.531	99.497	99.531	99.510
M3 (Graph features)	94.917	94.534	94.917	94.561
M4 (Quantum features)	99.313	99.259	99.313	99.046
Max_Fusion_M1_M2	99.541	99.517	99.541	99.506
Max_Fusion_M1_M3	99.360	99.326	99.360	99.179
Max_Fusion_M1_M4	99.361	99.346	99.361	99.236
Max_Fusion_M2_M3	99.551	99.504	99.551	99.509
Max_Fusion_M2_M4	99.549	99.516	99.549	99.508
Max_Fusion_M3_M4	99.348	99.360	99.348	99.218
Max_Fusion_M1_M2_M3	99.553	99.518	99.553	**99.518**
Max_Fusion_M1_M2_M4	99.547	99.517	99.547	99.514
Max_Fusion_M1_M3_M4	99.374	99.326	99.374	99.218
Max_Fusion_M2_M3_M4	99.553	99.518	99.553	99.514
**Max_Fusion_M1_M2_M3_M4**	**99.554**	**99.518**	**99.554**	99.516

**Table 9:** Detectability assessment across all datasets.

Class (Color-Coded) *	M1 TPR (%)	M2 TPR (%)	M3 TPR (%)	M4 TPR (%)	Best Model	Detectability
**DB1: NF-BoT-IoT**						
Benign (2.31%)	53.17	**76.33**	51.48	45.13	M2	N/A
Reconnaissance (78.43%)	93.18	93.08	**96.22**	92.89	M3	Supported
DDoS (9.47%)	11.76	44.69	32.05	**74.28**	M4	Partial Support
DoS (9.47%)	**85.79**	53.52	26.59	21.41	M1	Partial Support
Theft (0.32%)	71.47	**81.41**	59.16	59.69	M2	Partial Support
**DB2: NF-ToN-IoT**						
Benign (19.6%)	99.94	**99.99**	97.87	99.91	M2	N/A
Injection (33.97%)	88.15	78.38	82.27	**88.99**	M4	Not Supported
DDoS (23.66%)	79.94	**91.11**	89.70	82.16	M2	Supported
Password (11.33%)	**23.88**	12.55	4.01	11.37	M1	Not Supported
XSS (7.25%)	0.00	**20.73**	7.20	0.00	M2	Supported
Scanning (1.56%)	0.00	**35.47**	12.20	0.00	M2	Not Supported
DoS (1.28%)	0.00	0.00	**2.03**	0.00	M3	Not Supported
Backdoor (1.25%)	98.43	**99.30**	98.90	98.81	M2	Not Supported
MiTM (0.09%)	41.70	**74.52**	30.89	24.32	M2	Partial Support
Ransomware (0.01%)	0.00	**39.29**	0.00	0.00	M2	Partial Support
**DB3: NF-UNSW-NB15**						
Benign (95.54%)	99.45	**99.74**	99.34	99.59	M2	N/A
Exploits (1.52%)	78.51	78.59	**79.87**	78.21	M3	Partial Support
Fuzzers (1.20%)	33.21	**33.88**	24.61	13.02	M2	Partial Support
Reconnaissance (0.76%)	81.45	**96.34**	66.84	34.38	M2	Partial Support
Generic (0.34%)	24.51	**25.85**	12.48	18.85	M2	Supported
DoS (0.31%)	0.50	0.00	0.30	**0.89**	M4	Not Supported
Analysis (0.12%)	4.76	0.00	1.00	**12.28**	M4	Not Supported
Backdoor (0.11%)	0.00	0.00	2.25	**61.52**	M4	Partial Support
Shellcode (0.08%)	46.52	**96.70**	67.40	6.96	M2	Partial Support
Worms (0.01%)	0.00	0.00	0.00	0.00	None	Not Supported
**DB4: NF-CSE-CIC-IDS2018**						
Benign (87.86%)	**99.995**	99.836	98.537	99.981	M1	N/A
DDoS (4.53%)	99.916	**100**	61.231	99.933	M2	Supported
Brute Force (3.48%)	**99.971**	99.940	80.706	99.911	M1	Supported
DoS (3.21%)	**100**	**100**	76.609	**100**	M1, M2, M4	Supported
Infiltration (0.74%)	10.906	**57.584**	22.191	10.262	M2	Partial Support
Bot (0.19%)	**100**	**100**	76.602	**100**	M1, M2, M4	Not Supported
Injection (<0.01%)	0.0000	0.0000	0.0000	0.0000	None	Supported

* *Note: Grey = benign; green/yellow/red = high/medium/low detectability. Bold = best model per class.*

**Table 10:** Attack detectability assessment summary across datasets.

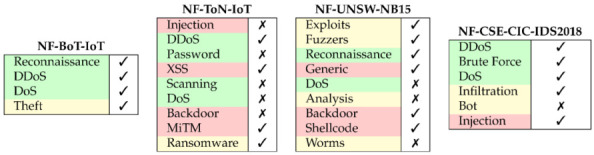

*Note: Cell colors indicate expected NetFlow detectability: green = high, yellow = medium, red = low. **✓** indicates that the hypothesis was supported; **✗** indicates that it was not supported.*

**Table 11:** Impact of varying number of transformer layers using FT-Transformer on NF-BoT-IoT.

Number of Transformer Layers	Accuracy (%)	Precision (%)	Recall (%)	F1-Score (%)
6	83.96	81.22	83.96	81.87
8	83.61	87.15	83.61	84.88
10	83.92	**87.50**	83.92	**84.91**
12	**83.99**	79.98	**83.99**	79.68
14	83.86	82.97	83.86	82.03

**Table 12:** Impact of varying learning rates using ELECTRA-Small on NF-BoT-IoT.

Learning Rate	Accuracy (%)	Precision (%)	Recall (%)	F1-Score (%)
$1\times 10^{-5}$	82.53	79.66	82.53	81.00
$2\times 10^{-5}$	83.45	81.76	83.45	80.87
$3\times 10^{-5}$	83.68	84.14	83.68	81.50
$4\times 10^{-5}$	**83.91**	**87.35**	**83.91**	**83.87**

**Table 13:** Impact of varying batch sizes using FT-Transformer on NF-BoT-IoT.

Batch Size	Accuracy (%)	Precision (%)	Recall (%)	F1-Score (%)
128	83.75	83.21	83.75	83.31
256	83.78	87.34	83.78	83.44
512	83.69	**87.63**	83.69	**84.10**
1024	83.94	80.16	83.94	80.67
2048	**84.04**	80.35	**84.04**	80.41

**Table 14:** Impact of varying number of epochs using ELECTRA-Small on NF-BoT-IoT.

Number of Epochs	Accuracy (%)	Precision (%)	Recall (%)	F1-Score (%)
1	82.39	80.70	82.39	81.47
3	83.61	85.37	83.61	83.64
5	**84.17**	**87.05**	**84.17**	**85.10**

**Table 15:** Impact of *k* on Node2Vec graph embedding performance on NF-ToN-IoT.

Number of *k* (Neighbors)	Accuracy (%)	Precision (%)	Recall (%)	F1-Score (%)
10	64.28	60.81	64.28	59.68
20	67.79	63.37	67.79	62.50
30	69.45	64.65	69.45	63.92
40	70.42	65.33	70.42	64.45
50	70.80	65.72	70.80	64.75
100	**71.05**	**66.21**	**71.05**	**65.55**

**Table 16:** Impact of varying quantum encoding dimensions on NF-UNSW-NB15.

Number of Qubits	Accuracy (%)	Precision (%)	Recall (%)	F1-Score (%)
10	96.42	94.90	96.42	95.62
12	96.49	95.27	96.49	95.60
14	96.68	95.92	96.68	96.00
16	**96.92**	96.31	**96.92**	96.40
18	96.91	**96.57**	96.91	**96.43**

## Data Availability

Data are contained within the article.

## Abbreviations

The following abbreviations are used in this manuscript:

BERT	Bidirectional Encoder Representations from Transformers
BiLSTM	Bidirectional Long Short-Term Memory
CIA	Confidentiality, Integrity, and Availability
CLS	Classification Token
CTI	Cyber Threat Intelligence
DDoS	Distributed Denial of Service
DL	Deep Learning
DoS	Denial of Service
DT	Decision Tree
ELECTRA	Efficiently Learning an Encoder that Classifies Token Replacements Accurately
FAISS	Facebook AI Similarity Search
FT-Transformer	Feature Tokenizer Transformer
GAN	Generative Adversarial Network
GNN	Graph Neural Network
GPT	Generative Pretrained Transformer
IDS	Intrusion Detection System
IoT	Internet of Things
IoV	Internet of Vehicles
IP	Internet Protocol
k-NN	k-Nearest Neighbors
L4/L7	Layer 4/Layer 7 (Transport/Application Layers)
LIME	Local Interpretable Model-Agnostic Explanations
LLM	Large Language Model
LSTM	Long Short-Term Memory
MiTM	Man-in-the-Middle
ML	Machine Learning
MLP	Multi-Layer Perceptron
MM-NIDS	Multimodal Network Intrusion Detection System
NF	NetFlow
NIST	National Institute of Standards and Technology
Node2Vec	Node to Vector
PCA	Principal Component Analysis
PEFT	Parameter-Efficient Fine-Tuning
PPO	Proximal Policy Optimization
RF	Random Forest
SHAP	Shapley Additive Explanations
SVM	Support Vector Machine
SYN	Synchronize (TCP Flag)
TCP	Transmission Control Protocol
UDP	User Datagram Protocol
ViT	Vision Transformer
XAI	Explainable Artificial Intelligence
XGBoost	Extreme Gradient Boosting
XSS	Cross-Site Scripting

## Appendix A. Additional Confusion Matrices

This appendix provides the additional confusion matrices that complement the representative example shown in Figure 5 for the NF-BoT-IoT dataset. Figure A1, Figure A2 and Figure A3 present the corresponding matrices for NF-ToN-IoT, NF-UNSW-NB15, and NF-CSE-CIC-IDS2018. Each figure includes four subfigures for the feature-specific base models (M1–M4). Rows indicate true labels, columns indicate predicted labels, and darker blue shading indicates higher classification frequency.
